# Coronary Artery Tortuosity With Coexisting Multiple Myocardial Bridges: A Cadaveric Case Report

**DOI:** 10.7759/cureus.115245

**Published:** 2026-08-26

**Authors:** Hannah J Grimmett, Victoria Comfort, Kamal A Abouzaid, Ahmad Imam, Ehab M Ishteiwy

**Affiliations:** 1 Department of Anatomical Sciences, William Carey University College of Osteopathic Medicine, Hattiesburg, USA

**Keywords:** case report, coronary artery variations, multiple myocardial bridges, severe coronary artery tortuosity, symmetrical pectus excavatum

## Abstract

Coronary artery tortuosity (CAT) and myocardial bridging are common anatomical variants often considered benign; however, both may alter coronary blood flow. Their extensive coexistence throughout the coronary arterial tree has rarely been reported. During routine cadaveric dissection of a 91-year-old female donor, severe diffuse CAT and multiple myocardial bridges were identified. The left anterior descending (LAD) artery, circumflex (Cx) artery, right coronary artery (RCA), posterior descending artery (PDA), acute marginal artery (AMA), and several major branches demonstrated marked tortuosity characterized by numerous looping and S-shaped configurations. The LAD demonstrated 11 bends; the AMA and PDA, eight each; the diagonal artery, seven; the circumflex artery, five; the obtuse marginal artery, four; and the proximal RCA, three. Four myocardial bridges were present: two over the LAD and one each over the AMA and PDA. Several looping segments appeared anchored to the myocardium by bridges or perforating branches. Despite the donor's advanced age, the coronary arteries exhibited no gross evidence of calcification or hardening. Additional findings included thin-walled translucent arterial segments and symmetrical pectus excavatum. This rare combination may have important clinical implications. The coexistence of myocardial bridges and CAT may alter coronary blood flow, while the associated pectus excavatum and translucent arterial walls may suggest an underlying connective tissue abnormality. Recognition of these complex coronary variations is important for accurate diagnosis, procedural planning, and surgical intervention.

## Introduction

Cardiovascular diseases remain the leading cause of death in the United States, with coronary artery disease being among the most prevalent heart conditions [1]. Coronary angiography is considered the gold standard for evaluating coronary anatomy and pathology [2]. Because coronary artery tortuosity (CAT) is a relatively common finding on coronary angiography, it is not typically documented by cardiologists [3].

Several studies have investigated the relationship between CAT and other patient characteristics. For example, Li et al. found tortuous coronary arteries to be significantly more common in women, while coronary artery disease and cigarette smoking were significantly associated with the lack of vessel tortuosity; this proposes the hypothesis that vessel tortuosity is potentially protective with respect to coronary artery disease [4]. Li et al. specifically investigated this relationship and found that tortuous segments of coronary vessels were significantly less likely to have atherosclerotic plaque formation [5].

Myocardial bridging (MB), which can be visually identified upon the removal of the pericardial fat, is defined as a segment of coronary artery that courses beneath an overlying band of myocardial fibers [6]. MB was first identified by Reyman in 1737 and then by Black in 1805; however, the first detailed post-mortem exam was conducted by Geiringer in 1951, with the first radiological description given in 1960 by Portmann and Iwig [7]. Nevertheless, in 1961, a landmark anatomical study examining 70 hearts ranging in age from newborn to 80 years old was conducted by Poláček. The researcher identified two different types of MB: areas where the artery submerges during its course along the ventricles and muscular loops attaching the artery to the atrial myocardium during its course in the atrioventricular groove; bridging was most frequently identified in the course of the left coronary artery (LCA). Interestingly, Poláček found that the portion of the artery proximal to the bridge or loop directly corresponds to the most common occlusion areas; he proposed that MB allows for a greater tendency for sclerotic plaque formation, thereby contributing to the development of an occlusion [6].

Reported incidence and prevalence rates of MB vary considerably depending on the diagnostic modality utilized. Recent studies using noninvasive imaging techniques have reported prevalence rates approaching 25%, with some studies documenting even higher rates [8]. Increased rates of MB have also been reported in patients with hypertrophic cardiomyopathy, suggesting a potential association between myocardial hypertrophy and the persistence of intramyocardial coronary segments [9]. The clinical significance of MB varies depending on the depth and location of the tunneled coronary artery. Consistent with the observations of Poláček, the left anterior descending (LAD) artery remains the coronary vessel most commonly affected by MB [6,8]. Although many cases remain asymptomatic, deeper myocardial encasement may result in systolic vessel compression associated with angina, thrombosis, myocardial infarction, and stress cardiomyopathy [8].

## Case presentation

During routine pedagogical cardiac dissection in the gross anatomy laboratory at William Carey University College of Osteopathic Medicine, severe CAT with concurrent MB was identified in a 91-year-old female cadaver. The donor, a Caucasian female obtained through the University of Alabama Anatomical Gift Program, had an unknown cause of death, and no clinical history was available.

**Figure 1:**
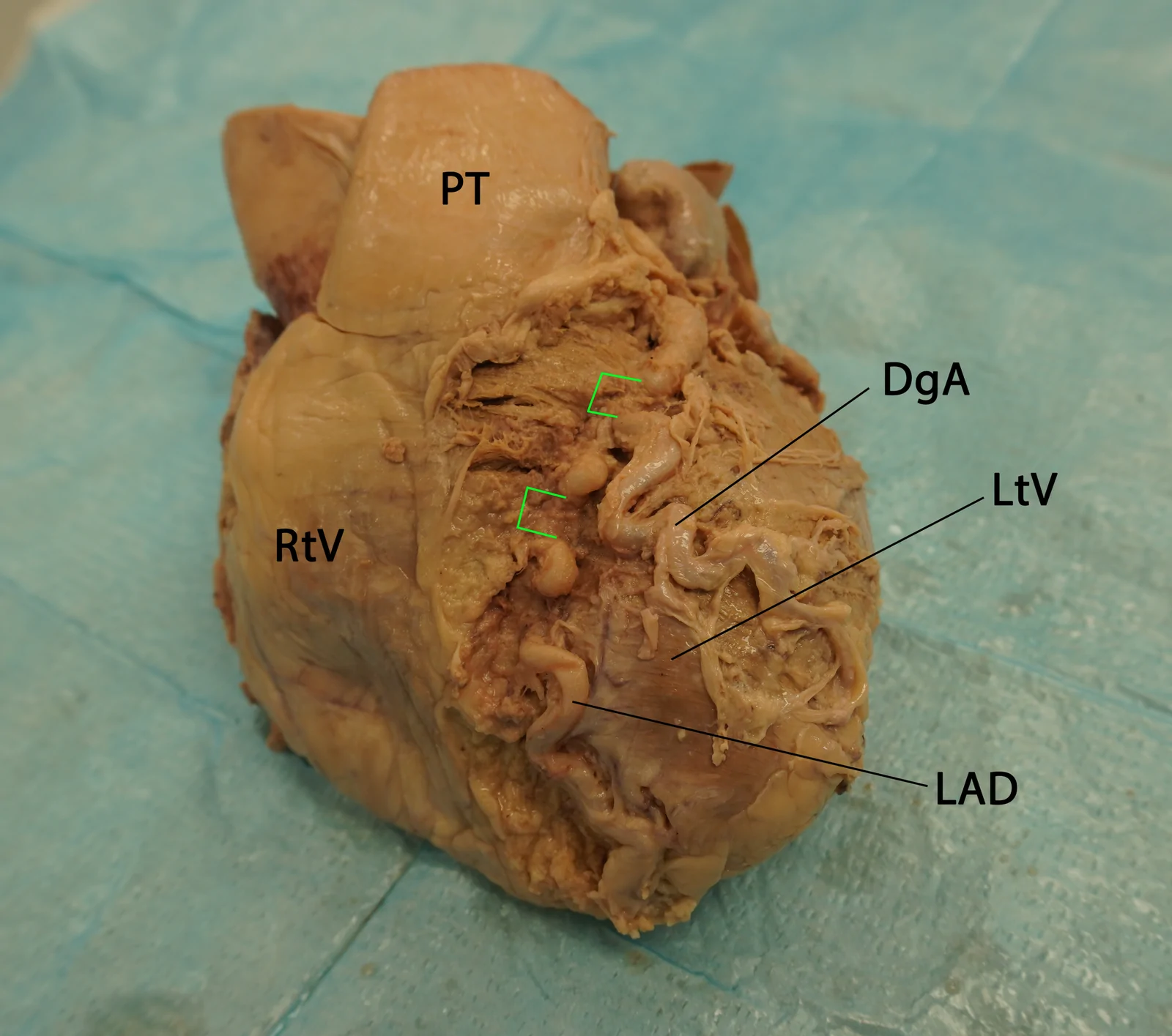
Anterior view of the heart demonstrating marked tortuosity of the LAD artery with its associated myocardial bridges and its diagonal branch along the anterior surface and obtuse margin of the heart. PT: Pulmonary Trunk, RtV: Right Ventricle, DgA: Diagonal Artery, LtV: Left Ventricle, LAD: Left Anterior Descending Artery, Green Brackets: Myocardial Bridges

The LCA originated from the left aortic sinus and coursed approximately 2.5 cm before bifurcating into the LAD artery and Cx artery. The LAD descended within the anterior interventricular sulcus following its expected anatomical course but demonstrated marked tortuosity, exhibiting a total of 11 bends before reaching the apex of the heart. A large diagonal branch arose from the LAD and was similarly tortuous, displaying seven bends along its course (Figure 1).

**Figure 2:**
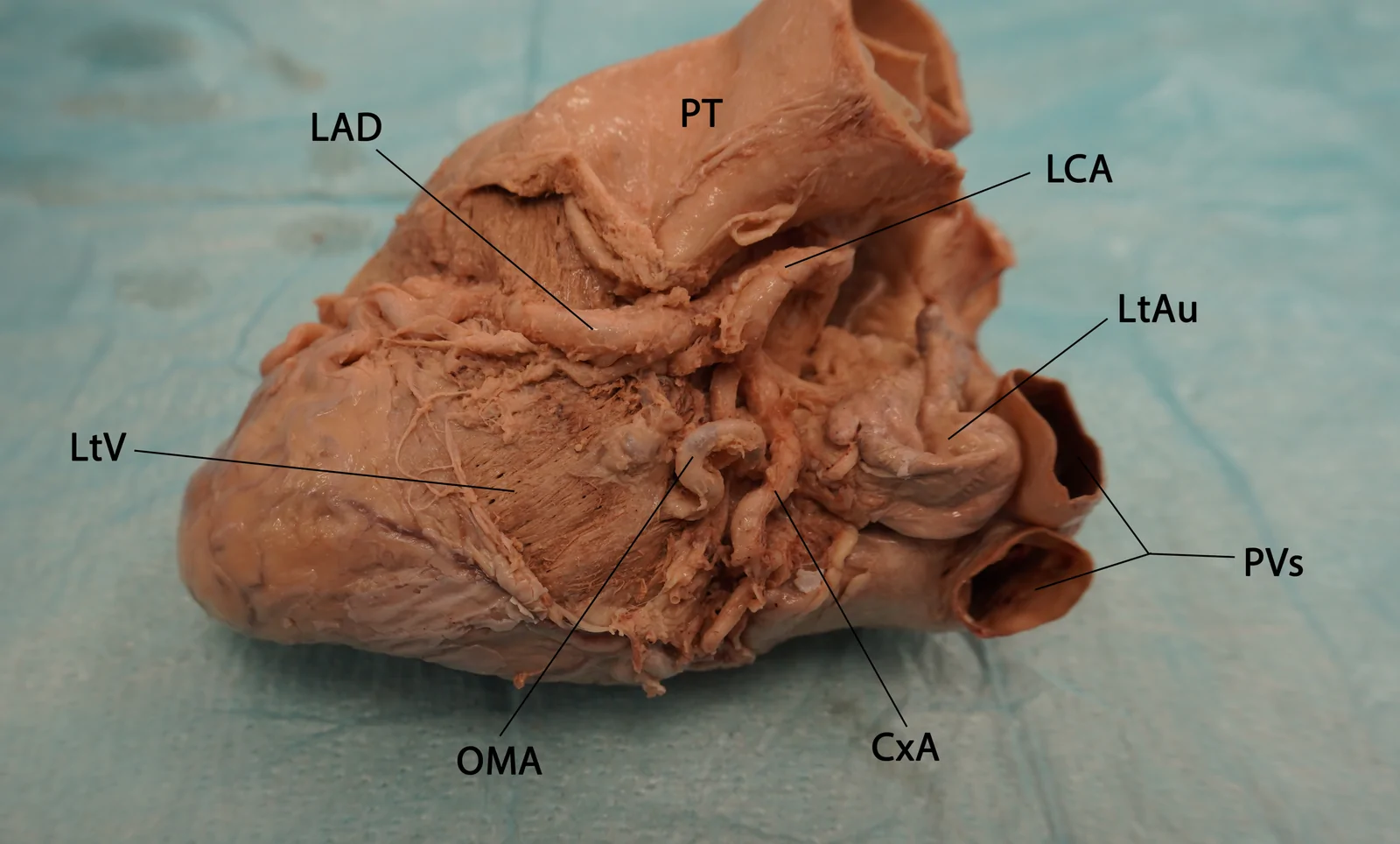
Left lateral view of the heart demonstrating the LCA, a tortuous Cx artery, and a tortuous OM branch. LtV: Left Ventricle, LAD: Left Anterior Descending Artery, OMA: Obtuse Marginal Artery, CxA: Circumflex Artery, LCA: Left Coronary Artery, LtAu: Left Auricle, PVs: Pulmonary Veins, PT: Pulmonary Trunk

The Cx artery traveled within the left atrioventricular groove and gave rise to a prominent obtuse marginal (OM) branch before continuing posteriorly and terminating through an anastomosis with the right coronary artery (RCA). The Cx artery exhibited five bends along its course. The OM branch descended along the obtuse margin of the left ventricle, displayed four bends, and subsequently entered the myocardium (Figure 2).

**Figure 3:**
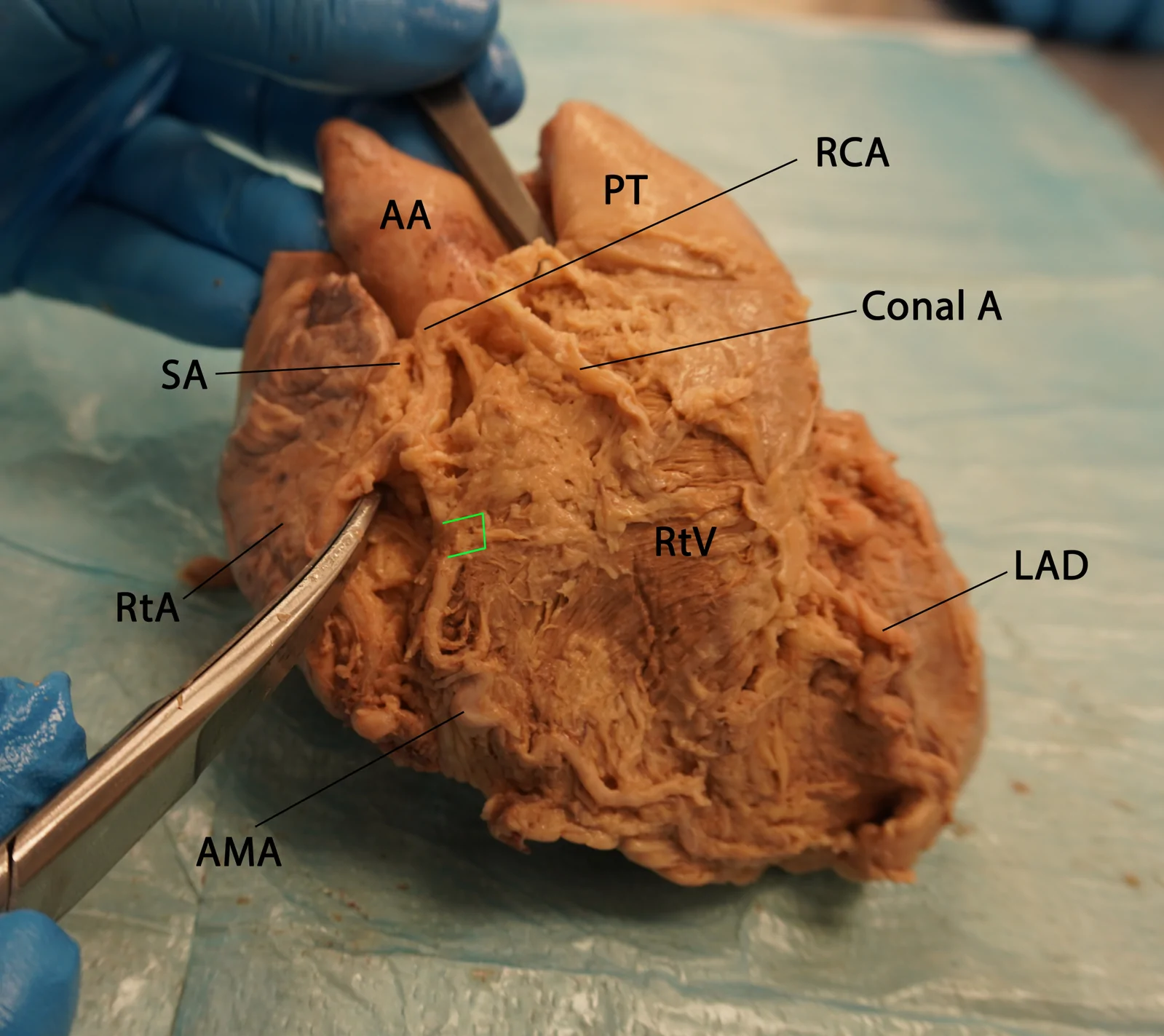
Right lateral view of the heart demonstrating the right coronary circulation, including a markedly tortuous AMA and an associated myocardial bridge crossing over the vessel. SA: Sinoatrial Nodal Branch, RtA: Right Atrium, AMA: Acute Marginal Artery, RtV: Right Ventricle, LAD: Left Anterior Descending Artery, Conal A: Conal Artery, RCA: Right Coronary Artery, PT: Pulmonary Trunk, AA: Ascending Aorta, Green Bracket: Myocardial Bridge

The RCA originated from the right aortic sinus and coursed within the right atrioventricular groove. Along its course, it gave rise to sinoatrial nodal, right atrial, conal, and acute marginal branches before continuing onto the diaphragmatic surface of the heart. The RCA demonstrated three bends before assuming a relatively straight course within the coronary sulcus. It then passed beyond the crux of the heart and continued between the left atrium and left ventricle, ultimately terminating through an anastomosis with the Cx artery. The acute marginal artery (AMA) displayed marked tortuosity with eight bends along its course around the acute margin of the heart (Figure 3).

**Figure 4:**
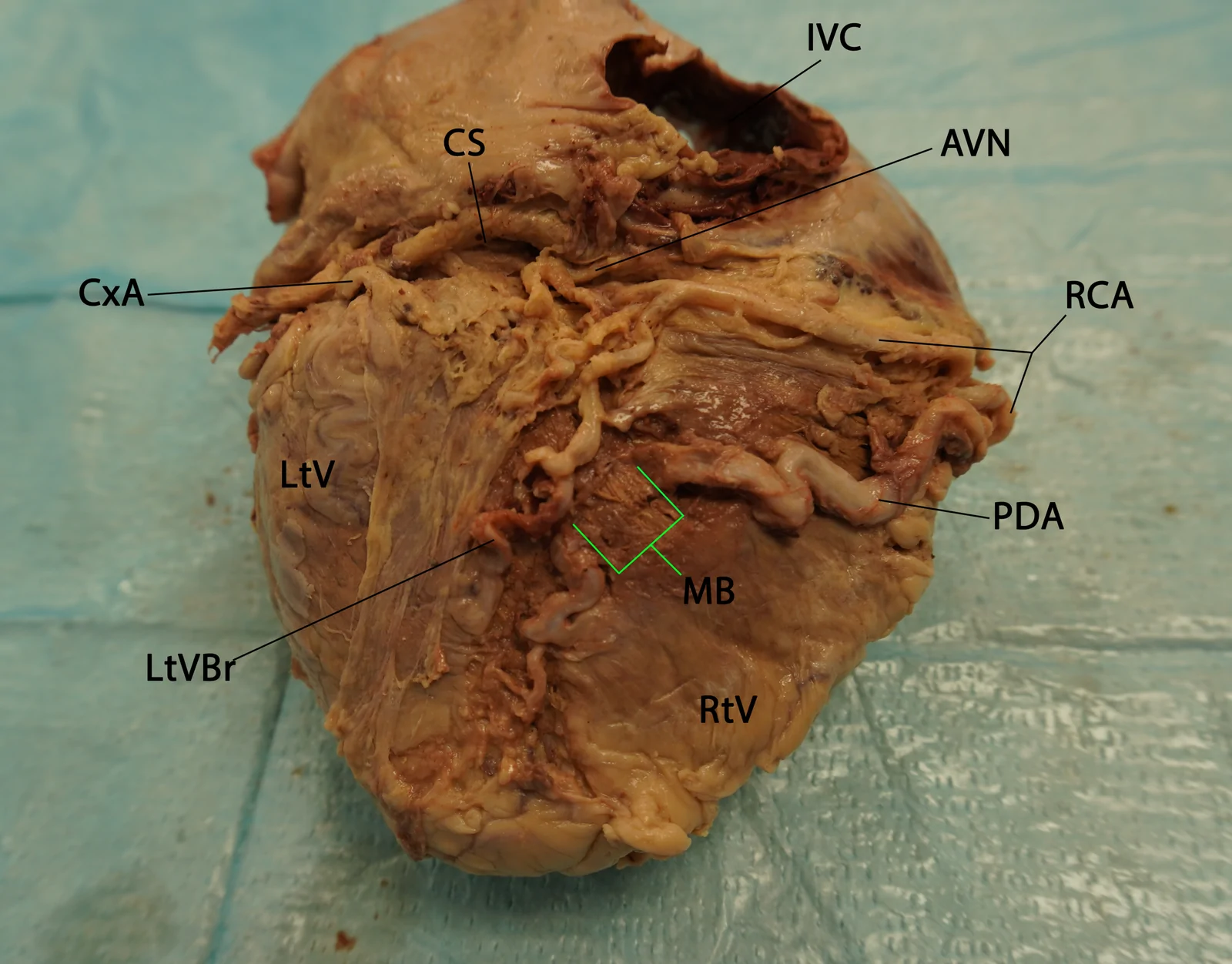
Inferior view of the heart demonstrating the diaphragmatic (posterior) surface and the right coronary circulation. Note the marked tortuosity of the PDA and an associated myocardial bridge crossing over the vessel. CxA: Circumflex Artery, LtV: Left Ventricle, LtVBr: Left Ventricular Branch, RtV: Right Ventricle, PDA: Posterior Descending Artery, RCA: Right Coronary Artery, AVN: Atrioventricular Nodal Branch, IVC: Inferior Vena Cava, CS: Coronary Sinus, Green Bracket/MB: Myocardial Bridge

On the diaphragmatic surface, the RCA gave rise to the posterior descending artery (PDA), which coursed obliquely toward the posterior interventricular sulcus and exhibited eight bends along its course. The atrioventricular nodal, left atrial, and left ventricular branches of the RCA were also identified. The left ventricular branch descended parallel to the PDA along the diaphragmatic surface of the heart, resembled an accessory PDA, and exhibited seven bends (Figure 4).

All major coronary arteries and their branches demonstrated marked tortuosity characterized by multiple looping configurations. These looping patterns were initially observed during dissection of the LCA and LAD and appeared as a series of inverted U-shaped loops. Further dissection revealed similar looping configurations throughout the coronary arterial tree. Collectively, the loops formed a succession of S-shaped vascular configurations. Some loops demonstrated broad U-turns, whereas others exhibited more acute angulations ranging from approximately 30° to 60°.

**Figure 5:**
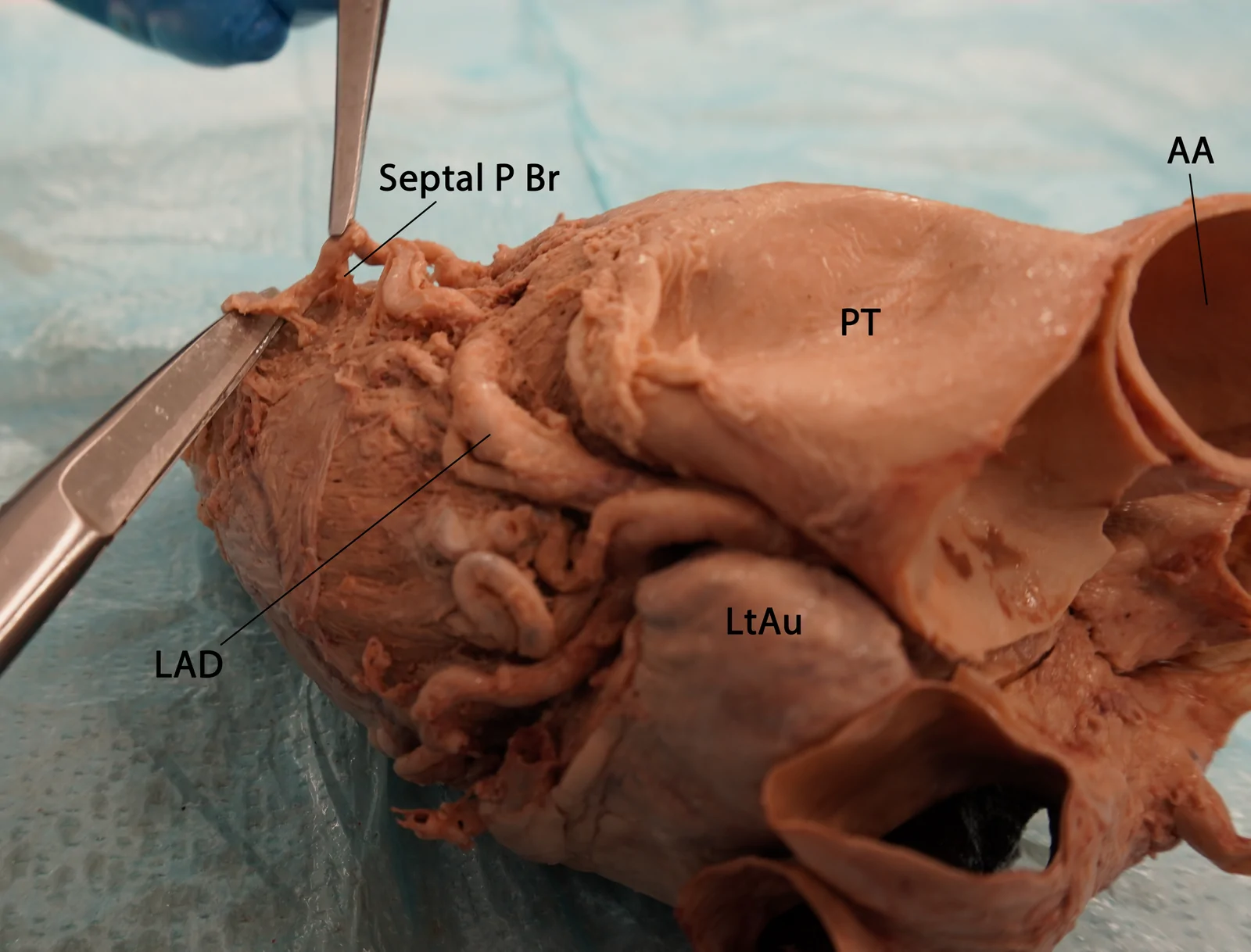
Superior (bird’s-eye) view of the left side of the heart demonstrating septal perforating branches. LAD: Left Anterior Descending Artery, LtAu: Left Auricle, PT: Pulmonary Trunk, AA: Ascending Aorta, Septal P Br: Septal Perforating Branches

Further examination revealed that the deeper looping segments were anchored to the myocardium either by myocardial perforating branches or by myocardial bridges (Figure 5).

**Figure 6:**
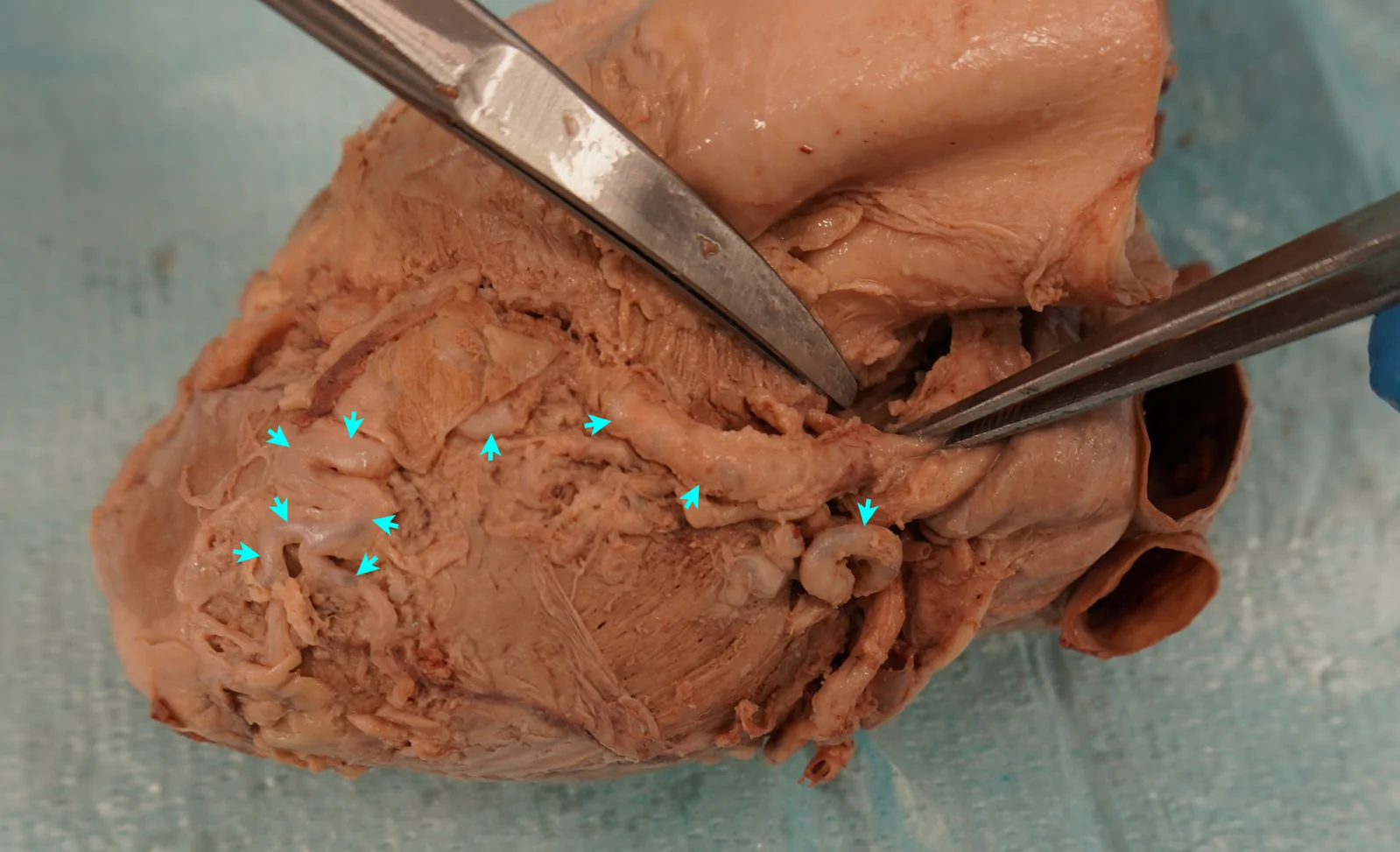
Left lateral view of the heart demonstrating several coronary arterial segments with unusually thin-walled and translucent appearances. Blue Arrowheads: Vessel Translucency

No gross calcification or appreciable hardening was identified upon palpation of the coronary arteries. One striking finding was that several of the looping arterial segments appeared thin-walled and translucent (Figure 6).

Multiple myocardial bridges were also identified. Two bridges were present over the LAD, one over the AMA, and one over the PDA. Proximal to the origin of the diagonal branch, a myocardial bridge was observed over the LAD, followed distally by a tortuous loop and a second myocardial bridge (Figure 1). The proximal LAD MB measured 8 mm in length and 6.5 mm in width, whereas the distal MB measured 5 mm in length and 5 mm in width. Both bridges were formed by grossly thin myocardial fibers.

The MB over the AMA measured 4 mm in length and 3.5 mm in width (Figure 3). Similar to the LAD bridges, it was composed of a thin layer of myocardial fibers. The myocardial bridge over the PDA measured 8 mm in length and 10 mm in width and appeared to be formed by a substantially thicker myocardial layer (Figure 4).

**Figure 7:**
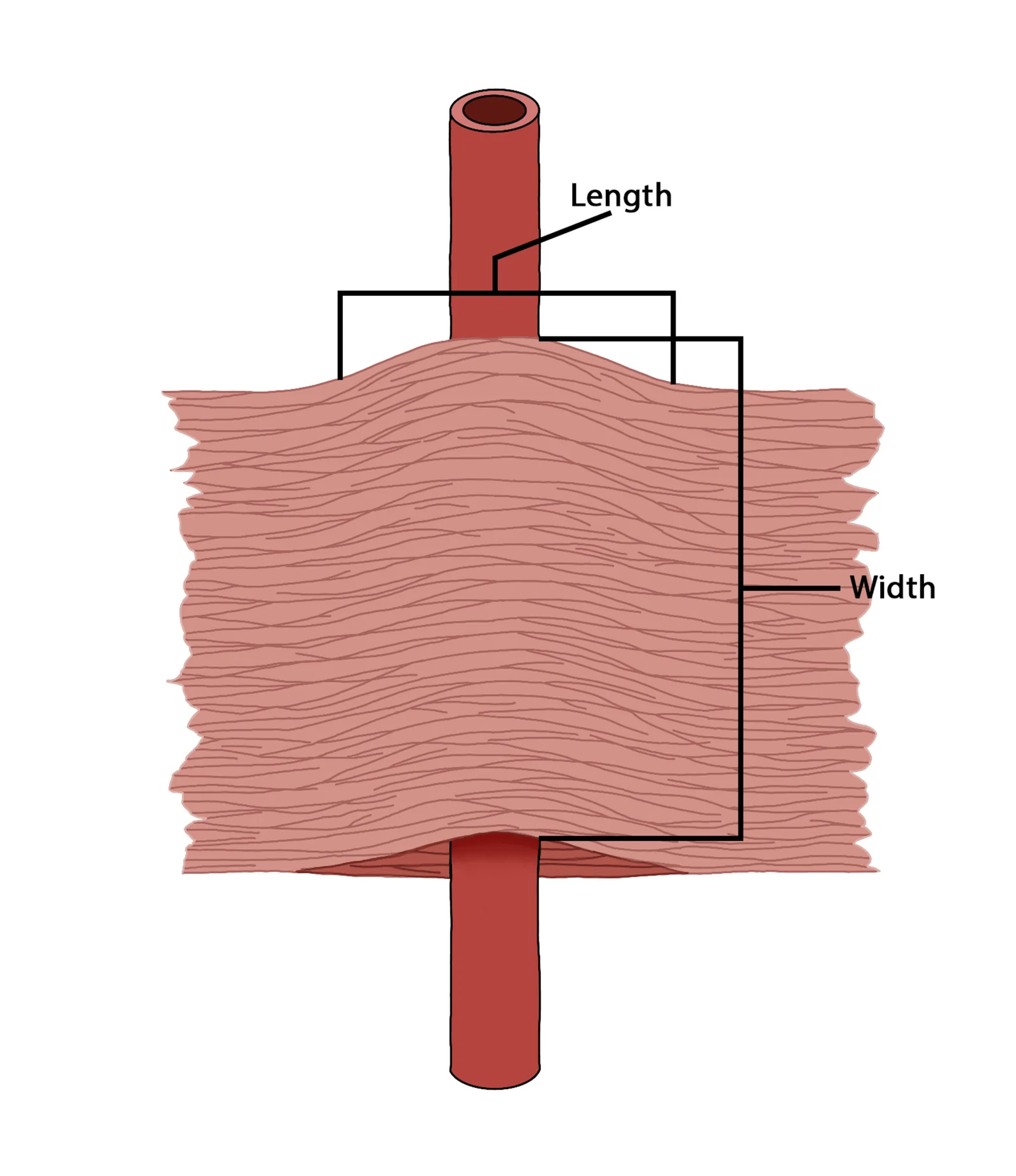
Schematic diagram illustrating the method used to measure myocardial bridge dimensions. This image was created and illustrated by Hannah Grimmett using Procreate (Savage Interactive Pty Ltd., Hobart, Australia).

The dimensions of each myocardial bridge were measured following careful removal of the overlying epicardial adipose tissue. Bridge length was defined as the distance along the course of the myocardial fibers extending from one side of the tunneled coronary artery to the other. Bridge width was defined as the distance between the proximal and distal margins of the myocardial bridge measured parallel to the long axis of the tunneled arterial segment (Figure 7).

Gross examination of the heart revealed normal cardiac size and overall morphology. No significant ventricular wall thinning, chamber dilation, valvular abnormalities, or calcific changes were observed. Internal examination of the cardiac chambers and valves demonstrated no obvious pathological findings.

**Figure 8:**
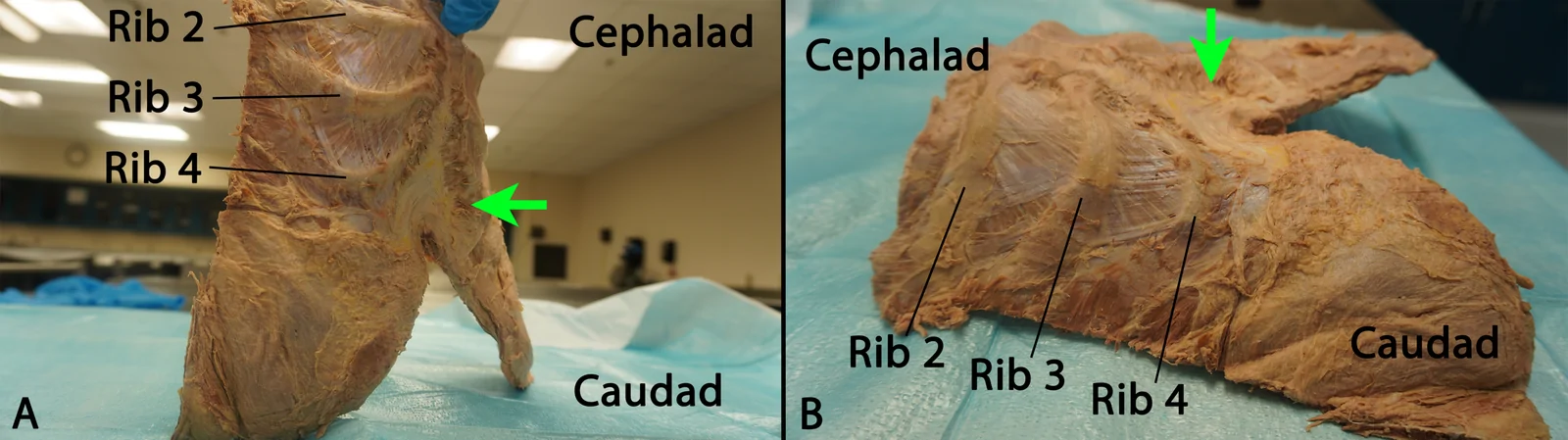
Right anterolateral views of the thoracic wall plate demonstrating pectus excavatum: (A) standing position and (B) supine position.

An additional noteworthy finding was the presence of symmetrical pectus excavatum. The sternal depression began at the level of the fourth sternocostal joints and involved the inferior portion of the sternum, with the deepest point located near the xiphoid process (Figure 8).

## Discussion

The present case demonstrates an unusual coexistence of severe diffuse CAT involving the entire coronary arterial tree with multiple myocardial bridges affecting the LAD, PDA, and AMA. Although CAT and MB are individually common anatomical variants, their concurrent occurrence in such an extensive distribution has rarely been reported. Furthermore, the tortuous vessels formed multiple looping configurations that were anchored by myocardial fibers and myocardial branches, creating a distinctive anatomical pattern not commonly described in the literature.

The coexistence of multiple myocardial bridges involving both the LAD and PDA has rarely been described in the literature. In a recent cadaveric case report, multiple myocardial bridges were identified over both the LAD and PDA [10]. These findings, together with prior reports describing symptomatic MB and its associated hemodynamic consequences, suggest that extensive myocardial encasement may alter coronary hemodynamics and contribute to ischemia, arrhythmias, and sudden cardiac death even in the absence of significant atherosclerotic disease [10-12]. However, unlike the previously reported case, the present specimen also demonstrated severe diffuse tortuosity involving all major coronary arteries and their branches, thereby representing a more complex anatomical variation.

Gaibazzi et al. investigated the presence of CAT and MB in patients with reversible myocardial perfusion defects despite angiographically normal coronary arteries. They found that individuals with either CAT or MB were more likely to report recurrent exertional angina and demonstrate ST-segment depression during treadmill exercise testing. These findings suggest that both anatomical variations may contribute to myocardial ischemia through mechanisms independent of atherosclerotic coronary artery disease [13].

Endocardiographic parameters have also been utilized to determine if vessel tortuosity is associated with structural and physiologic cardiac changes. Out of a cohort of 104, with 54 individuals having vessel tortuosity and 50 individuals lacking vessel tortuosity, peak early mitral inflow and the ratio of peak early mitral inflow to peak late mitral inflow were found to be significantly lower in individuals with tortuous coronary arteries; peak late mitral inflow, deceleration time of the peak early mitral inflow, isovolumic relaxation time, interventricular septal wall thickness at end-diastole, and left ventricular posterior wall thickness at end-diastole were found to be significantly higher in individuals with vessel tortuosity. Interestingly, no significant difference was noted with left ventricular ejection fraction when comparing the tortuous vessel group to the non-tortuous vessel group [2].

Several pathological conditions have been associated with CAT. One of the most notable associations is fibromuscular dysplasia (FMD). Saw et al. reported some degree of coronary tortuosity in every patient included in their study of FMD [14]. Additionally, FMD has also been strongly associated with spontaneous coronary artery dissection (SCAD), a recognized cause of acute coronary syndrome in patients without significant atherosclerotic disease. In a cohort of 246 patients with SCAD, 78% demonstrated tortuous coronary arteries [15].

Little research has been done investigating conditions associated with both MB and tortuous coronary arteries. Takotsubo cardiomyopathy, however, serves as a prime example where the aforementioned variations have been observed. Takotsubo cardiomyopathy, also referred to as apical ballooning syndrome, is a known, reversible cause of ACS [16]. Zegers et al. identified three cases of patients suffering from ACS who were found to have both observable cardiac wall defects and tortuous coronary arteries [17]. Likewise, Migliore et al. examined patients diagnosed with Takotsubo cardiomyopathy and noted a high prevalence of MB among the patient population [16]. These studies suggest that both anatomical variants may influence coronary flow patterns and potentially contribute to the pathogenesis of the disease.

The translucent appearance and thin-walled character of several coronary arterial segments, together with the presence of pectus excavatum in this case, may be suggestive of an underlying connective tissue disorder. Al Fadley et al. evaluated a group of patients who were referred for either a known cardiac murmur or atypical facial features. All of these patients were found to have hyperextensibility of the skin and hypermobility of all joints, but they lacked the cutaneous cardinal features found in Ehlers-Danlos; additionally, no abnormal findings were noted on light electron microscopy studies of the skin. While these patients had various abnormalities noted throughout their circulatory system, all demonstrated significant CAT; this, in conjunction with their atypical phenotypes, suggests either collagen or elastin could be involved in the development of the tortuous vessels [18].

Recognition of these coronary artery variations is important because both may influence diagnostic and therapeutic procedures. Increased vessel tortuosity has been associated with greater technical difficulty and increased complication rates during percutaneous coronary interventions, although it is not considered a contraindication to intervention [19]. Similarly, patients with symptomatic MB are typically managed medically with pharmacologic therapy and antiplatelet agents, while persistent symptoms may require percutaneous intervention, surgical myotomy, or coronary artery bypass grafting [8,20]. Therefore, accurate recognition of these anatomical variants is essential for procedural planning and risk assessment.

## Conclusions

This case documents a rare coexistence of CAT involving the entire coronary arterial tree with multiple MBs affecting both the LAD and PDA, accompanied by distinctive looping arterial segments anchored by myocardial fibers and myocardial branches. The extensive distribution and unique anatomical relationship of these variations may have important clinical implications. Awareness of such complex coronary anatomy is essential for accurate diagnostic assessment, procedural planning, and surgical intervention. This case expands the existing anatomical literature and highlights the need for further investigation into the developmental, physiological, and clinical significance of concurrent CAT and MB.
